# Local Tendon Transfers for Chronic Ruptures of the Achilles Tendon: A Systematic Review

**DOI:** 10.3390/jcm12020707

**Published:** 2023-01-16

**Authors:** Nicola Maffulli, Salvatore Ziello, Gianluca Maisto, Filippo Migliorini, Francesco Oliva

**Affiliations:** 1 Department of Musculoskeletal Disorders, Faculty of Medicine and Surgery, University of Salerno, 84084 Baronissi, Italy; 2 Clinica Ortopedica, Ospedale San Giovanni di Dio e Ruggi d’Aragona, 84131 Salerno, Italy; 3 Centre for Sports and Exercise Medicine, Barts and the London School of Medicine and Dentistry, Queen Mary University of London, Mile End Hospital, 275 Bancroft Road, London E1 4DG, UK; 4 School of Pharmacy and Bioengineering, Keele University Faculty of Medicine, Thornburrow Drive, Stoke-on-Trent ST4 7QB, UK; 5 Department of Orthopaedic, Trauma, and Reconstructive Surgery, RWTH University Hospital, 52074 Aachen, Germany

**Keywords:** Achilles tendon, tendon transfer, mid-portion Achilles’ chronic ruptures

## Abstract

Introduction: A rupture of the Achilles tendon with a delay in diagnosis or treatment for more than 6 weeks is considered a chronic tear. Local tendon transfer procedures can be used in chronic Achilles tendon ruptures. This study evaluated the outcome, return to sport, and complications of local tendon transfer in patients with chronic Achilles tendon rupture. Material and methods: The present study was conducted according to the PRISMA 2020 guidelines. PubMed, Google Scholar, Embase, and Web of Science databases were accessed in November 2022. Results: Data were retrieved from 23 articles (463 patients, mean age 50.9 ± 13.5 years). The mean duration of the follow-up was 58.3 ± 76.8 months. The VAS improved by 1.8/10 (*p* = 0.4), the AOFAS by 33.4/100 (*p* < 0.0001), and the ATRS by 32.5/100 (*p* = 0.0001) points. Patients were able to return to sport after a mean of 19.6 ± 16.4 weeks. A total of 79% of patients were able to return to their previous activities. The rate of complications was 13.3%. Conclusions: The use of local tendon transfer for chronic Achilles tendon ruptures using the FHL or PB tendon resulted in good clinical outcomes and a reliable return to daily activities and sports. The rate of complications reflects the chronicity of the condition and the technical complexity of the procedure. Level of evidence: IV.

## 1. Introduction

Classically located within 2–7 cm of its distal insertion on the calcaneus [1], tears of the Achilles tendon (AT), the strongest tendon in the human body [2], are frequently not diagnosed in a timely fashion, and a rupture with a delay in diagnosis or treatment for more than 6 weeks is considered a chronic tear [1].

Tendon transfer procedures can be used in Achilles tendinopathy and chronic ruptures [3,4]. Various techniques have been described for AT reconstruction and augmentation, including local tendon transfers (flexor hallucis longus, peroneus brevis, flexor digitorum longus, and peroneus longus) and grafts (autograft, allograft, and synthetic graft) [5].

The transfer of the flexor hallucis longus (FHL) tendon was first described by Hansen in 1991 [6]. Several modifications have been described since, including single or double incisions, short or long transfer, different methods of fixation, and an open or endoscopic approach, and it is now the most commonly published technique to reconstruct a chronic tear [4]. This procedure has several advantages: the FHL tendon is easy to harvest given its proximity to the AT and can be harvested with small incisions, minimizing the risk of neurovascular injury and wound healing complications [4]. Transfer of the peroneus brevis (PB) tendon was popularized by Perez-Teuffer in 1974 [7]. PB is suitable for patients with a tendon gap inferior to 6 cm and has a low rate of wound healing complications [8]. The transfer of the flexor digitorum longus (FDL) tendon was described by Mann et al. in 1991 [9]. It is an alternative in the treatment of these injuries, with low complications and donor site morbidity [10]. Transfers of the peroneus longus (PL) tendon have also been reported but are less commonly used in clinical practice [11]. The choice of the optimal technique is still debated, and no consensus has been reached. The present study analyzes in a systematic fashion the differences between the various local tendon transfers in the management of chronic Achilles tendon ruptures, evaluating clinical outcomes, complications, and return to sport.

## 2. Material and Methods

### 2.1. Search Strategy

This systematic review was conducted according to the Preferred Reporting Items for Systematic Reviews and Meta-Analyses: the 2020 PRISMA statement [12]. It was registered on the International Prospective Register of Systematic Reviews (PROSPERO; Registration No. CRD42022384213).

### 2.2. Eligibility Criteria

All the prospective and retrospective studies reporting local tendon transfer for the management of chronic Achilles tendon tears were accessed. According to the authors language capabilities, articles in English, Italian, German, and Spanish were eligible. Reviews, opinions, letters, and editorials were not considered. Animal, biomechanics, computational, and cadaveric studies were not eligible.

The PICOT algorithm was preliminarily pointed out:

- P (Problem): Chronic rupture of the mid-portion Achilles tendon;
- I (Intervention): Transfer;
- C (Comparison): FHL, PB, FDL, and PL tendon transfers;
- O (Outcomes): Clinical outcomes, complications, and return to sport;
- T (Timing): ≥6 months of follow-up.

In November 2022, PubMed, Web of Science, Google Scholar, and Embase databases were accessed. No time constraints were used for the search. The following keywords were used in combination: Achilles tendon, tendon transfer, mid-portion Achilles chronic ruptures, mid portion Achilles rupture, main body Achilles rupture, main body Achilles chronic rupture.

### 2.3. Selection and Data Collection

Two authors independently performed the database search. All the resulting titles were screened, and if suitable, the abstracts were accessed. The full text of the abstracts that matched the topic of interest was accessed. The bibliography of the full-text articles was also screened by hand to identify other eligible articles for inclusion. Disagreements were debated, and the final decision was made by a third senior author.

### 2.4. Methodological Quality Assessment

Two authors independently performed the methodological quality assessment using the Coleman Methodology Score (CMS). The CMS is a 10 item scale designed to rate the methodological quality of the included studies [13]. These items evaluated study size, mean follow-up, number of surgical procedures, type of study, diagnostic certainty, description of surgical procedure, postoperative rehabilitation, outcome measures, outcome assessment, and selection process. The final score ranges between 0 and 100, with a score of 100 indicating the highest reported methodological quality [13]. (Table 1 and Table 2).

### 2.5. Data items

Two authors independently performed data extraction. The following data were extracted: generalities (author, year, and type of study), demographic baseline (number of samples and mean age), mean follow-up, mean BMI, and surgical intervention (FHL transfer and PB transfer). The primary outcome of interest was the clinical outcome: the Visual Analogue Scale (VAS), the American Orthopaedic Foot and Ankle Society (AOFAS), and the Achilles tendon Total Rupture Score (ATRS). The secondary outcome of interest was complications. The third outcome of interest was a return to sports.

### 2.6. Outcomes

The scales used to evaluate clinical outcome were the VAS, AOFAS, and ATRS.

In 1921, Hayes and Patterson introduced the Visual Analogue Scale (VAS) as a pain rating scale [34], measuring the frequency and intensity of pain. It consists of a 10 cm line, the left end of which represents a state of “no pain”, while the right one represents “the worst pain”. The patients mark on the line the point that matches their perception of their current state [35].

The American Orthopaedic Foot and Ankle Society (AOFAS) Ankle-Hindfoot Score combined a clinical reported (developed by Kitaoka et al. in 1994 [36]) and a patient reported part to evaluate the outcome of treatment in patients with ankle or hindfoot injury. This rating system is divided into three categories: pain (40 points), function (50 points), and alignment (10 points), with nine questions in each for a total of 100 points. Zero stands for severe pain or impairment; 100 for no pain. Though widely used, the AOFAS score has not been validated.

The Achilles tendon Total Rupture Score (ATRS) measures the outcome related to symptoms and physical activity after treatment in patients with total AT rupture. The scale ranged from 0 = major limitations/symptoms to 100 = no limitations/symptoms and has now been cross-culturally validated in several languages [37]. To date, only the ATRS is a condition-specific PROM.

### 2.7. Statistical Analysis

The statistical analysis was performed using IBM SPSS version 25. Mean and standard deviation were used for descriptive statistics. For continuous variables, the mean difference effect measure was used. The paired *t*-test was used with values of *p* < 0.05 considered statistically significant.

## 3. Results

### 3.1. Study Selection

**Figure 1:**
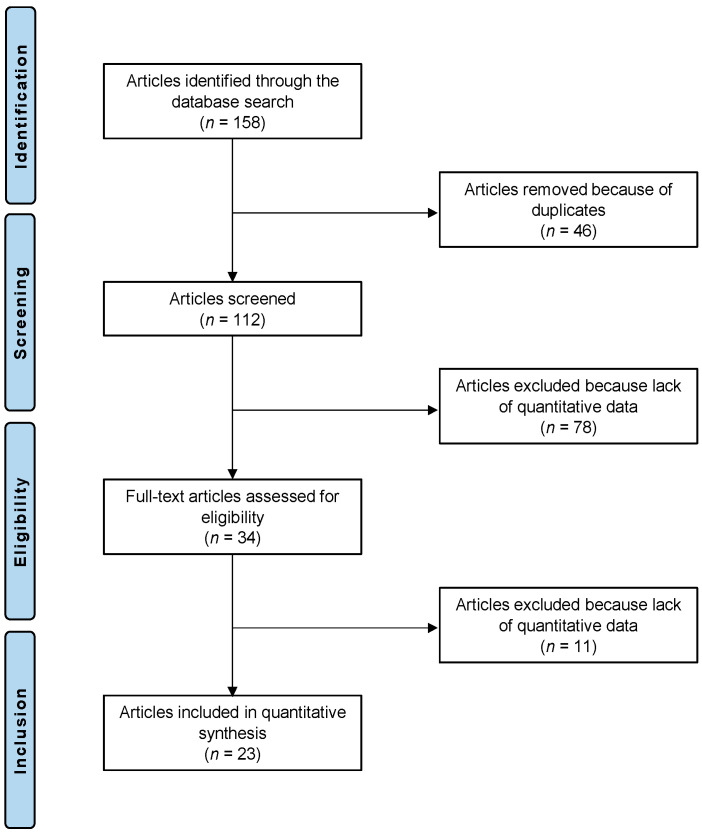
PRISMA flow diagram of the present systematic review.

The initial literature search resulted in 158 studies. Of them, 46 were excluded as being duplicates. Another 78 were not eligible: not matching the topic (*n* = 63), focusing on surgical technique with no outcome data (*n* = 8), type of study (*n* = 3), full text not accessible (*n* = 2), or uncertain results (*n* = 2). This left 34 articles for inclusion. An additional 11 studies were excluded as they did not report quantitative data under the outcomes of interest. This resulted in 23 studies left for analysis. We placed our main focus on the FHL and PB transfers since studies on other transfers matching the inclusion criteria for the current study had not been carried out. The results of the literature search are shown in Figure 1.

### 3.2. Study Risk of Bias Assessment

The length of follow-up was acceptable in most studies. Surgical technique, diagnosis, and rehabilitation protocols were generally well described. The size of the study and the retrospective design of most of the included studies represented the main limitations highlighted by the CMS. Outcome measures, assessment timing, and selection processes were also clearly defined by most studies. Finally, the mean Coleman Methodology Score of 73.8 (range: 59–85) attests to the overall good quality of the methodological assessment (Table 1 and Table 2).

### 3.3. Study Characteristics and Results of Individual Studies

A total of 463 patients were identified; 25.1% (120 of 463) were females. The mean length of the follow-up was 58.3 ± 76.8 months. The mean age was 50.9 ± 13.5 years, and the mean BMI was 26.8 ± 0.9 kg/m^2^. The generalities of the included studies are shown in Table 3.

### 3.4. Results of Syntheses

All the patient-reported outcome measures (PROMs) significantly improved at the last follow-up (Table 4). The VAS reduced by 1.8/10 (*p* = 0.4), the AOFAS improved by 34.3/100 (*p* < 0.0001), and the ATRS improved by 41.3/100 (*p* = 0.0001) (Table 4).

Calf circumference did not improve significantly (*p* = 0.08). Patients were able to return to their daily activities at a mean of 13.7 ± 8.3 weeks and to sports at a mean of 19.6 ± 16.4 weeks. A total of 79% of patients were able to return to practice the previous activity. The overall rate of complications was 12.7% (59 complications in 463 procedures) (Table 5).

## 4. Discussion

Several local tendon transfers have been described, but the tendons most commonly used in transfers for chronic ruptures of the Achilles tendon are those of the FHL and PB. 

The transfer of the flexor hallucis longus (FHL) tendon is the most reported, carrying, at least theoretically, a series of advantages over other local tendon transfers:

- The FHL is the second strongest plantar flexor muscle of the ankle;
- Its axis of action is in line with that of the AT;
- It maintains normal ankle muscle balance;
- Its harvest carries a low risk of iatrogenic neurovascular injury;
- It increases the vascularity of the reconstruction given its low-lying muscle belly [32].

A potential undesired effect of FHL harvest is the loss of plantar flexion of the interphalangeal joint of the hallux, with decreased plantar flexion and push-off strength [5]. However, despite the weakness of plantar flexion of the hallux, most patients do not report noticeable deformities or weakness and resume their pre-injury daily activities [14]. 

The morbidity associated with FHL tendon transfer seems not to be clinically relevant, even in running sports that require good push-off or balance [5]. 

In the present systematic review, the rate of complications following the use of FHL transfers is 14.8%. Of the 338 patients, the major complications were one deep vein thrombosis, four deep infections, and one re-rupture [17] (Table 5).

The tendon of the peroneus brevis (PB) is well vascularized. The transfer allows the blood supply from the musculotendinous junction to be maintained, providing a robust reinforcement to the AT [38].

The two peroneal muscles contribute only 4% of the capacity for plantar flexion, while the PB tendon contributes approximately 28% of the total eversion strength [38]. Intuitively, the use of the PB tendon may cause a strength deficit in eversion of the ankle and not affect plantar flexion [30]. The peroneus longus is the major evertor of the hindfoot, and it may take over some of the functions of the PB, reducing subjective weakness in ankle function after PB tendon transfer [39]. Human cadaveric models have been used to assess the mechanical properties of AT reconstruction with the PB and FHL [40]. The outcomes were similar in terms of stiffness (16.5 ± 6.3 N/mm (PB) vs. 14.0 ± 3.8 N/mm (FHL)), energy to failure (3656.0 ± 2720.3 J (PB) vs. 2406.7 ± 1621.8 J (FHL)), and mode of failure. The force to failure of the PB tendon transfer was higher compared to the FHL (348.8 ± 124.9 N (PB) vs. 241.5 ±8 2.2 N (FHL)), and this difference was statistically significant, although it may not be clinically relevant. The authors of [40] reported good clinical results in the use of PB, with a final ATRS of 92.5 associated with a low rate of complications. In fact, patients did not experience deep vein thrombosis nor re-rupture, and five superficial infections were managed with oral antibiotics [8].

The present systematic review reported a rate of complications in the use of PB tendon transfer of 7% in 128 patients, five of whom experienced superficial infections and four wound complications (Table 5). Due to the lack of relevant data, it was not possible to directly compare the complications of FHL and PB or to analyse which surgical technique leads to a higher rate of complications.

The return to daily activities and the return to sport were only reported in three and four studies, respectively; seventy-nine and ninety-nine patients were evaluated; patients were able to return to daily activities in 13.7 weeks and return to sport in 19.6 weeks. Maffulli et al. reported a slower return to sport in PB tendon transfer patients compared to FHL transfer patients, but a higher percentage of PB transfer patients eventually returned to sport compared to FHL transfer patients [25].

The use of flexor digitorum longus (FDL) tendon transfer has been described in two reports; however, these were excluded since they described its use in the management of Achilles tendinopathy. The FDL tendon transfer is an alternative operative technique that keeps the FHL tendon intact, preserves push-off strength, and minimizes gait disturbances [10]. However, De Cesar Netto et al. reported a patient with weakness of plantar flexion of the lesser toes, without balance or gait disturbances [10]. In addition to the transfer of the tendon of the FDL, they also performed a turndown of the central third of the proximal aspect of the AT or a hamstring allograft reconstruction [10].

The peroneal longus (PL) tendon has a stronger failure load than the tendon of the PB [11]. However, the use of the PL tendon may cause a long-lasting strength deficit in eversion of the ankle, especially at a higher angular velocity, more evident than in the transfer of the PB [38]. 

This study has several limitations. First, the retrospective design and the lack of blinding in most of the included studies. Given the lack of quantitative data available for inclusion, it was not possible to analyze the results of each transfer separately. Moreover, we excluded several studies because most of them did not separate data on patients with chronic AT rupture from patients with Achilles tendinopathy and acute AT rupture, while others did not differentiate mid-portion ruptures from ruptures of the insertion of the AT. Furthermore, the inhomogeneity of the evaluation scales prevents an adequate comparison between the studies. Given the lack of relevant quantitative data, further subgroup analyses were not possible. Further investigations are required to validate the results of the present study in a clinical setting.

## 5. Conclusions

The use of local tendon transfer for chronic Achilles tendon ruptures using the FHL or PB tendon showed good clinical outcomes and allowed a reliable return to daily activities and sports. Better-quality future studies are needed to ascertain which surgical procedures are most advantageous for these patients.

## Figures and Tables

**Table 1:** Methodological Quality Assessment: Coleman Methodological Score.

Authors, Years	Part A: Only One Score to Be Given for Each of the 7 Sections						
	Study Size	Mean Follow-Up	Surgical Approach	Type of Study	Description of Diagnosis	Descriptions of Surgical Technique	Description of Postoperative Rehabilitation
Abubeih et al., 2018 [14]	4	4	10	10	5	10	5
Ahn et al., 2022 [15]	4	7	10	0	5	10	5
Alauddinet al., 2022 [16]	4	0	10	10	5	5	0
Alhaug et al., 2019 [17]	4	7	10	0	5	10	5
Elias et al., 2007 [18]	4	4	10	0	5	10	5
Khalid et al., 2019 [19]	0	4	10	0	5	10	5
Koh et al., 2019 [20]	4	4	0	0	5	10	5
Lever et al., 2018 [21]	4	10	10	0	5	10	5
Lui et al., 2012 [22]	0	7	10	10	5	10	0
Maffulli et al., 2010 [8]	4	7	10	10	5	10	5
Maffulli et al., 2012 [23]	4	10	10	10	5	10	5
Maffulli et al., 2015 [24]	4	7	10	0	5	10	5
Maffulli et al., 2018 [25]	4	4	7	10	5	10	5
Mahajan et al., 2009 [26]	4	4	10	0	5	10	5
Miao et al., 2016 [27]	4	4	10	0	5	10	5
Oksanen et al., 2014 [28]	0	4	10	0	5	10	5
Ozer et al., 2018 [29]	4	10	10	10	5	10	5
Pintore et al., 2001 [30]	7	7	7	10	5	10	5
Singh et al., 2014 [31]	4	4	10	0	5	10	5
Tay et al., 2010 [32]	0	4	10	10	5	10	5
Vega et al., 2018 [33]	4	4	10	0	5	10	5
Wegrzyn et al., 2010 [5]	0	10	10	0	5	10	5
Yeoman et al., 2012 [34]	0	0	10	10	5	10	5

**Table 2:** Methodological Quality Assessment: Coleman Methodological Score.

Authors, Years	Part B: Scores May Be Given for Each Option in Each of the Three Sections If Applicable											Total
	Outcome Criteria				Procedure Used to Assess Outcomes				Description of the Subject Selection Process			
	Outcome Measures Clearly Defined	Timing of Outcome Assessment Clearly Stated	Use of Outcome Criteria That Have Reported Reliability	General Health Measure Included	Participants Recruited	Investigator Independent of Surgeon	Written Assessment	Completion of Assessment by Patients Themselves with Minimal Investigator Assistance	Selection Criteria Reported and Unbiased	Recruitment Rate Reported > 80%	Recruitment Rate Reported < 80%	
Abubeih et al., 2018 [14]	2	2	3	0	5	4	3	3	5	5	0	80
Ahn et al., 2022 [15]	2	2	3	3	5	4	3	3	5	5	0	76
Alauddin et al., 2022 [16]	2	2	3	0	5	0	0	3	5	5	0	59
Alhaug et al., 2019 [17]	2	2	3	0	5	4	3	3	5	5	0	73
Elias et al., 2007 [18]	2	2	3	0	5	4	3	3	5	5	0	70
Khalid et al., 2019 [19]	2	2	3	0	5	4	3	3	5	5	0	66
Koh et al., 2019 [20]	2	2	3	0	5	4	3	3	5	5	0	60
Lever et al., 2018 [21]	2	2	3	0	5	4	3	3	5	5	0	76
Lui et al., 2012 [22]	2	2	3	0	5	4	3	3	5	5	0	74
Maffulli et al., 2010 [8]	2	2	3	0	5	0	3	3	5	5	0	79
Maffulli et al., 2012 [23]	2	2	3	0	5	0	3	3	5	5	0	82
Maffulli et al., 2015 [24]	2	2	3	3	5	4	3	3	5	5	0	76
Maffulli et al., 2018 [25]	2	2	3	3	5	0	3	3	5	5	0	76
Mahajan et al., 2009 [26]	2	2	3	3	5	4	3	3	5	5	0	73
Miao et al., 2016 [27]	2	2	3	3	5	4	3	3	5	5	0	73
Oksanen et al., 2014 [28]	2	2	3	3	5	4	3	3	5	0	0	64
Ozer et al., 2018 [29]	2	2	3	3	5	0	3	3	5	5	0	85
Pintore et al., 2001 [30]	2	2	3	3	5	0	3	3	5	5	0	82
Singh et al., 2014 [31]	2	2	3	3	5	4	3	3	5	5	0	73
Tay et al., 2010 [32]	2	2	3	3	5	4	3	3	5	5	0	79
Vega et al., 2018 [33]	2	2	3	3	5	4	3	3	5	5	0	73
Wegrzyn et al., 2010 [5]	2	2	3	3	5	4	3	3	5	5	0	75
Yeoman et al., 2012 [34]	2	2	3	3	5	4	3	3	5	5	0	75

**Table 3:** Generalities and patient baseline databases.

Author et al., Year	Journal Name	Design	Technique	Follow-Up (Months)	Patients (*n*)	Age (Mean)	Female (*n*)
Abubeih et al., 2018 [14]	*Int Orthop*	Prospective	open FHL	15	21	40.3	6
Ahn et al., 2022 [15]	*J Foot Ankle Surg*	Retrospective	open FHL	57	28	51	11
Alauddin et al., 2022 [16]	*Mymensingh Med J*	Prospective	open FHL	6	21	39.5	
Alhaug et al., 2019 [17]	*Foot Ankle Surg*	Retrospective	open FHL	54	21	54.5	6
Elias et al., 2007 [18]	*Foot Ankle Int*	Retrospective	open FHL	24.4	15	55.8	5
Khalid et al., 2019 [19]	*Foot Ankle Spec*	Retrospective	endoscopic FHL	30.9	10	58.4	5
Koh et al., 2019 [20]	*Foot Ankle Surg*	Retrospective	open FHL	12	29	56	13
Lever et al., 2018 [21]	*Bone Joint J*	Retrospective	open FHL	73	20	53	4
Lui et al., 2012 [22]	*Foot Ankle Spec*	Prospective	endoscopic FHL	37	5	46	2
Maffulli et al., 2010 [8]	*Am J Sports Med*	Prospective	open PB	48.4	32	47.13	4
Maffulli et al., 2012 [23]	*J Bone Joint Surg Am*	Prospective	open PB	186	16	55.6	0
Maffulli et al., 2015 [24]	*Bone Joint J*	Retrospective	mini-open PB	55.2	17	39	3
Maffulli et al., 2018 [25]	*Foot Ankle Surg*	Prospective	mini-open FHL	35.8	21	42.7	9
			mini-open PB	36.4	20	45.8	6
Mahajan et al., 2009 [26]	*J Orthop Surg*	Retrospective	open FHL	12	36	70	12
Miao et al., 2016 [27]	*Indian J Orthop*	Retrospective	mini-open FHL	32.2	32	42.1	14
Oksanen et al., 2014 [28]	*Foot Ankle Surg*	Retrospective	open FHL	27	7	53	3
Ozer et al., 2018 [29]	*J Foot Ankle Surg*	Prospective	open FHL	280	19	47.4	1
Pintore et al., 2001 [30]	*J Trauma*	Prospective	open PB	53	21	43.3	1
Singh et al., 2014 [31]	*J Orthop Surg*	Retrospective	mini-open PB	12	22	28	
Tay et al., 2010 [32]	*Ann Acad Med Singap*	Prospective	open FHL	24	6	59.5	
Vega et al., 2018 [33]	*Foot Ankle Int*	Retrospective	endoscopic FHL	30.5	22	69	6
Wegrzyn et al., 2010 [5]	*Int Orthop*	Retrospective	open FHL	79	11	44	4
Yeoman et al., 2012 [34]	*Foot (Edinb)*	Prospective	open FHL	6	11	52.6	5

FHL: flexor hallucis longus; PB: p eroneus brevis.

**Table 4:** Patient-reported outcome measures.

Endpoint	Baseline	Last Follow-Up	Mean Deviation	*p*-Value
VAS	2.6 ± 0.6	0.8 ± 0.8	−1.8	0.04
AOFAS hindfoot	57.1 ± 8.5	91.4 ± 4.7	34.3	<0.0001
ATRS	44.4 ± 19.2	85.7 ± 7.5	41.3	<0.0001

VAS: Visual Analogue Scale; AOFAS hindfoot: American Orthopaedic Foot and Ankle Society hindfoot; ATRS: Achilles tendon Total Rupture Score.

**Table 5:** Complications.

Complications	FHL (338 Procedures)			PB (128 Procedures)	
	Open	Mini-Open	Endoscopic	Open	Mini-Open
Pain			2		
Superficial infection	11			5	
Deep infection	4				
Deep venous thrombosis	1				
Focal numbness	4				
Wound complications	9	1	1	2	
Scar adhesion	1				
Weak push-off	3				
Hypertrophic scarring of the incision				2	
Re-rupture	1				
Claw toes	2				
Reduced skin sensation	6				
Neurological complications	4				

## Data Availability

Not applicable.

## Abbreviations

AOFAS	American Orthopaedic Foot and Ankle Society
AT	Achilles tendon
ATRS	Achilles tendon Total Rupture Score
BMI	Body Mass Index
CMS	Coleman Methodology Score
FDL	flexor digitorum longus
FHL	flexor hallucis longus
IBM SPSS	International Business Machines Statistical Package for the Social Sciences
PB	peroneus brevis
PICOT	Problem, Intervention, Comparison, Outcomes, Timing
PL	peroneous longus
PRISMA	Preferred Reporting Items for Systematic Reviews and Meta-Analyses
PROMs	patient-reported outcome measures
VAS	Visual Analogue Scale

## References

[1] Maffulli N. (1999). Rupture of the Achilles tendon. J. Bone Jt. Surg. Am..

[2] Viidik A. (1969). Tensile strength properties of Achilles tendon systems in trained and untrained rabbits. Acta Orthop. Scand..

[3] Hahn F., Meyer P., Maiwald C., Zanetti M., Vienne P. (2008). Treatment of chronic achilles tendinopathy and ruptures with flexor hallucis tendon transfer: Clinical outcome and MRI findings. Foot Ankle Int..

[4] Yassin M., Gupta V., Martins A., Mahadevan D., Bhatia M. (2021). Patient reported outcomes and satisfaction following single incision Flexor Hallucis Longus (FHL) augmentation for chronic Achilles tendon pathologies. J. Clin. Orthop. Trauma.

[5] Wegrzyn J., Luciani J.F., Philippot R., Brunet-Guedj E., Moyen B., Besse J.L. (2010). Chronic Achilles tendon rupture reconstruction using a modified flexor hallucis longus transfer. Int. Orthop..

[6] Hansen S.T. (1991). Trauma to the heel cord. Disorders of the Foot and Ankle. Med. Surg. Manag..

[7] Perez Teuffer A. (1974). Traumatic rupture of the Achilles Tendon. Reconstruction by transplant and graft using the lateral peroneus brevis. Orthop. Clin. N. Am..

[8] Maffulli N., Spiezia F., Longo U.G., Denaro V. (2010). Less-invasive reconstruction of chronic achilles tendon ruptures using a peroneus brevis tendon transfer. Am. J. Sports Med..

[9] Mann R.A., Holmes G.B., Seale K.S., Collins D.N. (1991). Chronic rupture of the Achilles tendon: A new technique of repair. J. Bone Jt. Surg. Am..

[10] de Cesar Netto C., Chinanuvathana A., Fonseca L.F.D., Dein E.J., Tan E.W., Schon L.C. (2019). Outcomes of flexor digitorum longus (FDL) tendon transfer in the treatment of Achilles tendon disorders. Foot Ankle Surg..

[11] Wang C.C., Lin L.C., Hsu C.K., Shen P.H., Lien S.B., Hwa S.Y., Pan R.Y., Lee C.H. (2009). Anatomic reconstruction of neglected Achilles tendon rupture with autogenous peroneal longus tendon by EndoButton fixation. J. Trauma.

[12] Page M.J., McKenzie J.E., Bossuyt P.M., Boutron I., Hoffmann T.C., Mulrow C.D., Shamseer L., Tetzlaff J.M., Akl E.A., Brennan S.E. (2021). The PRISMA 2020 statement: An updated guideline for reporting systematic reviews. BMJ.

[13] Coleman B.D., Khan K.M., Maffulli N., Cook J.L., Wark J.D. (2000). Studies of surgical outcome after patellar tendinopathy: Clinical significance of methodological deficiencies and guidelines for future studies. Scand. J. Med. Sci. Sport. Rev. Artic..

[14] Abubeih H., Khaled M., Saleh W.R., Said G.Z. (2018). Flexor hallucis longus transfer clinical outcome through a single incision for chronic Achilles tendon rupture. Int. Orthop..

[15] Ahn J., Jeong B.O. (2022). Return to Sports Activities after Flexor Hallucis Longus Transfer for Neglected Achilles Tendon Rupture. J. Foot Ankle Surg..

[16] Alauddin M., Hossain M.Z., Rahman M.M., Roy M.K., Minto M.R., Islam M.A., Islam M.K., Islam M.S., Saha M.K., Mahmud A.A. (2022). Management of Neglected Rupture of Tendoachilles with Long Gap by Flexor Hallucis Longus Tendon Transfer. Mymensingh Med. J..

[17] Alhaug O.K., Berdal G., Husebye E.E., Hvaal K. (2019). Flexor hallucis longus tendon transfer for chronic Achilles tendon rupture. A retrospective study. Foot Ankle Surg..

[18] Elias I., Besser M., Nazarian L.N., Raikin S.M. (2007). Reconstruction for missed or neglected Achilles tendon rupture with V-Y lengthening and flexor hallucis longus tendon transfer through one incision. Foot Ankle Int..

[19] Khalid M.A., Weiss W.M., Iloanya M., Panchbhavi V.K. (2019). Dual Purpose Use of Flexor Hallucis Longus Tendon for Management of Chronic Achilles Tendon Ruptures. Foot Ankle Spec..

[20] Koh D., Lim J., Chen J.Y., Singh I.R., Koo K. (2019). Flexor hallucis longus transfer versus turndown flaps augmented with flexor hallucis longus transfer in the repair of chronic Achilles tendon rupture. Foot Ankle Surg..

[21] Lever C.J., Bosman H.A., Robinson A.H.N. (2018). The functional and dynamometer-tested results of transtendinous flexor hallucis longus transfer for neglected ruptures of the Achilles tendon at six years’ follow-up. Bone Jt. J..

[22] Lui T.H. (2012). Treatment of chronic noninsertional Achilles tendinopathy with endoscopic Achilles tendon debridement and flexor hallucis longus transfer. Foot Ankle Spec..

[23] Maffulli N., Spiezia F., Pintore E., Longo U.G., Testa V., Capasso G., Denaro V. (2012). Peroneus brevis tendon transfer for reconstruction of chronic tears of the Achilles tendon: A long-term follow-up study. J. Bone Jt. Surg. Am..

[24] Maffulli N., Oliva F., Costa V., Del Buono A. (2015). The management of chronic rupture of the Achilles tendon: Minimally invasive peroneus brevis tendon transfer. Bone Jt. J..

[25] Maffulli N., Oliva F., Maffulli G.D., Buono A.D., Gougoulias N. (2018). Surgical management of chronic Achilles tendon ruptures using less invasive techniques. Foot Ankle Surg..

[26] Mahajan R.H., Dalal R.B. (2009). Flexor hallucis longus tendon transfer for reconstruction of chronically ruptured Achilles tendons. J. Orthop. Surg..

[27] Miao X., Wu Y., Tao H., Yang D., Huang L. (2016). Reconstruction of Kuwada grade IV chronic achilles tendon rupture by minimally invasive technique. Indian J. Orthop..

[28] Oksanen M.M., Haapasalo H.H., Elo P.P., Laine H.J. (2014). Hypertrophy of the flexor hallucis longus muscle after tendon transfer in patients with chronic Achilles tendon rupture. Foot Ankle Surg..

[29] Ozer H., Ergisi Y., Harput G., Senol M.S., Baltaci G. (2018). Short-Term Results of Flexor Hallucis Longus Transfer in Delayed and Neglected Achilles Tendon Repair. J. Foot Ankle Surg..

[30] Pintore E., Barra V., Pintore R., Maffulli N. (2001). Peroneus brevis tendon transfer in neglected tears of the Achilles tendon. J. Trauma.

[31] Singh A., Nag K., Roy S.P., Gupta R.C., Gulati V., Agrawal N. (2014). Repair of Achilles tendon ruptures with peroneus brevis tendon augmentation. J. Orthop. Surg..

[32] Tay D., Lin H.A., Tan B.S., Chong K.W., Rikhraj I.S. (2010). Chronic Achilles tendon rupture treated with two turndown flaps and flexor hallucis longus augmentation—Two-year clinical outcome. Ann. Acad. Med. Singap..

[33] Vega J., Vilá J., Batista J., Malagelada F., Dalmau-Pastor M. (2018). Endoscopic Flexor Hallucis Longus Transfer for Chronic Noninsertional Achilles Tendon Rupture. Foot Ankle Int..

[34] Yeoman T.F., Brown M.J., Pillai A. (2012). Early post-operative results of neglected tendo-Achilles rupture reconstruction using short flexor hallucis longus tendon transfer: A prospective review. Foot.

[35] Delgado D.A., Lambert B.S., Boutris N., McCulloch P.C., Robbins A.B., Moreno M.R., Harris J.D. (2018). Validation of Digital Visual Analog Scale Pain Scoring with a Traditional Paper-based Visual Analog Scale in Adults. J. Am. Acad. Orthop. Surg. Glob. Res. Rev..

[36] Kitaoka H.B., Alexander I.J., Adelaar R.S., Nunley J.A., Myerson M.S., Sanders M. (1994). Clinical rating systems for the ankle-hindfoot, midfoot, hallux, and lesser toes. Foot Ankle Int..

[37] Nilsson-Helander K., Thomeé R., Silbernagel K.G., Thomeé P., Faxén E., Eriksson B.I., Karlsson J. (2007). The Achilles tendon Total Rupture Score (ATRS): Development and validation. Am. J. Sports Med..

[38] Clarke H.D., Kitaoka H.B., Ehman R.L. (1998). Peroneal tendon injuries. Foot Ankle Int..

[39] Gallant G.G., Massie C., Turco V.J. (1995). Assessment of eversion and plantar flexion strength after repair of Achilles tendon rupture using peroneus brevis tendon transfer. Am. J. Orthop..

[40] Sebastian H., Datta B., Maffulli N., Neil M., Walsh W.R. (2007). Mechanical properties of reconstructed achilles tendon with transfer of peroneus brevis or flexor hallucis longus tendon. J. Foot Ankle Surg..

